# Who's Afraid of the Boss: Cultural Differences in Social Hierarchies Modulate Self-Face Recognition in Chinese and Americans

**DOI:** 10.1371/journal.pone.0016901

**Published:** 2011-02-16

**Authors:** Sook-Lei Liew, Yina Ma, Shihui Han, Lisa Aziz-Zadeh

**Affiliations:** 1 The Brain and Creativity Institute, University of Southern California, Los Angeles, California, United States of America; 2 Division of Occupational Science and Occupational Therapy, University of Southern California, Los Angeles, California, United States of America; 3 Department of Psychology, Peking University, Beijing, People's Republic of China; University of Maribor, Slovenia

## Abstract

Human adults typically respond faster to their own face than to the faces of others. However, in Chinese participants, this self-face advantage is lost in the presence of one's supervisor, and they respond faster to their supervisor's face than to their own. While this “boss effect” suggests a strong modulation of self-processing in the presence of influential social superiors, the current study examined whether this effect was true across cultures. Given the wealth of literature on cultural differences between collectivist, interdependent versus individualistic, independent self-construals, we hypothesized that the boss effect might be weaker in independent than interdependent cultures. Twenty European American college students were asked to identify orientations of their own face or their supervisors' face. We found that European Americans, unlike Chinese participants, did not show a “boss effect” and maintained the self-face advantage even in the presence of their supervisor's face. Interestingly, however, their self-face advantage decreased as their ratings of their boss's perceived social status increased, suggesting that self-processing in Americans is influenced more by one's social status than by one's hierarchical position as a social superior. In addition, when their boss's face was presented with a labmate's face, American participants responded faster to the boss's face, indicating that the boss may represent general social dominance rather than a direct negative threat to oneself, in more independent cultures. Altogether, these results demonstrate a strong cultural modulation of self-processing in social contexts and suggest that the very concept of social positions, such as a boss, may hold markedly different meanings to the self across Western and East Asian cultures.

## Introduction

“At home, a young man should be dutiful towards his parents; going outside, he should be respectful towards his elders.”

-Confucius (Chinese philosopher, 551–479 BC)

“Your real boss is the one who walks under your hat.”

-Napoleon Hill (American author, 1833–1970)

Cultural differences play a key role, not only in how people understand themselves, but also in how they relate to others. This is exemplified in the quotations above, with the former Chinese quote emphasizing the importance of respecting one's elders both at home and in public while the latter American one affirms one's independence and autonomy above all else. Several decades of both behavioral and neuroimaging research suggest that self-concept is largely determined by one's culture, with notable differences between East Asian and Western cultures [1]–[6]. In particular, people from Western countries tend to be more individualistic and have what is known as an *independent self-construal* [5]. In these cases, the self is thought of as an isolated unit that strives to be unique, autonomous, and assertive, functioning in parallel with, but not dependent upon, others. In contrast, those from more collectivist cultures, such as East Asians, tend to demonstrate an *interdependent self-construal*, in which the self is conceptualized in terms of its relationship to others, which blurs the distinction between self and other and allows the self to be easily modulated by dynamic social contexts, such as the presence of one's supervisor [5].

There are a significant number of findings that attribute differences in both cognitive processes and affective states to these noted cultural differences in self-construals [1]–[8]. For instance, individuals with independent self-construals tend to be more assertive and use competitive conflict tactics in group work settings, while individuals with interdependent self-construals are more likely to shy away from conflict and use cooperative tactics [6]. In addition, the interdependent self-construal was positively correlated with ease of embarrassment while the independent self-construal was negatively correlated; Asian Americans were more easily embarrassed than European Americans [7]. Neuroimaging studies have also shown that while Americans activate neural regions associated with self-processing (e.g., the medial prefrontal cortex) only when thinking about oneself, Chinese participants activate these self-processing regions both when thinking about oneself *and* one's close family members, like one's mother [8]. Similarly, an EEG study showed that images of one's own face, compared to familiar faces, elicited greater fronto-central activity, related to self-processing, in British participants but less fronto-central activity in Chinese [9], demonstrating the blurred distinction between self and other in Chinese individuals. In addition, when looking across cultures, neural activity in the mPFC was predictive of how individualistic or collectivist participants were [1]. These results suggest that interdependent individuals are much more affected by social contexts than independent individuals, and that interdependent self-construals encompass other individuals while independent self-construals largely include only the self.

Cultural differences in self-construals also affect relationships in work environments, where individuals must navigate complex social hierarchies. In line with the idea of independent self-construals, Americans are generally encouraged to be socially dominant, competitive, and assertive, while East Asians tend to value subordinance, cooperation, and harmony [10]–[12]. Weisz et al. (1984) elaborate on these differences as desiring primary control (e.g., social dominance, as found typically in Americans) versus secondary control (e.g., social subordination, as found typically in East Asians), and note that these cultural differences affect a myriad of social activities including work, child-rearing, and religious involvement. A recent neuroimaging study provided support for these findings by demonstrating that Americans show neural activity in reward-related brain regions in response to signals of dominance, while Japanese participants show neural activity in these same reward-related brain regions in response to signals of subordination [13]. In addition, self-construal appears to play a role in mediating social interactions. One study found that the higher self-esteem an individual has, the more strongly he or she demonstrates positive self-protective behaviors in response to negative feedback from others [14]. However, this was true only in American participants and in Chinese participants who demonstrated a more independent self-construal. Chinese participants who were more interdependent did not demonstrate self-protective behaviors in relation to negative feedback, suggesting that one's self-construal affects how one interprets and reacts to social threats.

The self-construal has been studied in a number of ways, with a wealth of literature suggesting that one's own face is even processed differently from faces of others [15]–[19]. Behavioral studies show faster reaction times (RTs) to one's own face compared to another's face during either explicit face–recognition tasks requiring judgments of face identity [15], [16] or implicit face recognition tasks requiring determination of whether a face is oriented to the right or left [18]. Notably, these effects are most significant on left-hand responses, leading researchers to suggest that this is reflective of self-processing, which is thought to occur in the right hemisphere [15], [16], [18], [20]. Ma and Han (2010) [18] suggest that this self-face RT advantage may be due to implicit positive associations with the self. In a series of 4 experiments, they demonstrated that self-concept threat priming (i.e., deciding whether negative trait words describe oneself) weakens one's implicit positive associations with oneself and decreases the self-face RT advantage, an effect that is seen in left-hand (but not right-hand) responses [18]. These interesting results suggest that threats to one's self-concept, which weaken one's implicit positive association with oneself, may reduce any advantages in self-referential processing.

A recently discovered phenomenon, known as the “boss effect,” supports these findings. Ma and Han (2009) found that Chinese graduate students demonstrated a typical self-face advantage (i.e., faster RT to one's own face than another's face) when their faces were presented in a block with a familiar faculty member's face [20]. However, when their faces were presented with their boss's face, participants lost the self-face advantage and demonstrated significantly faster RTs to their boss's face, which the authors termed the “boss effect” [20]. Notably, participants did not demonstrate significant RT differences when shown blocks with faces of their boss and a labmate, suggesting that the boss effect was specific to the social threat incurred when one's own face was paired with the presence of one's boss.

The current study assessed whether the “boss effect” on self-face recognition is culturally universal or specific to cultures dominated by interdependent self-construals. We hypothesized that this boss effect might be modulated by cultural influences on participants' self-construals. In support of this, Ma and Han (2010) found that while similar effects of self-concept threat are observed in both Chinese and American participants, they occurred to a much lesser extent in Americans as compared to Chinese [18]. In addition, Sui et al. (2009) found that event related potentials recorded from British participants showed larger amplitudes to self-face than to a friend's face whereas a reverse pattern was observed in Chinese participants [9], suggesting greater social salience of self-face in people with independent-self construals compared to those with interdependent self-construals. Thus, here we surmised that more independent selves would be less affected by social contexts and hierarchies, and therefore less susceptible to any social threat induced by seeing one's boss. We anticipated that American participants would demonstrate a self-face advantage in all contexts, whether paired with their boss (high social threat) or another faculty member (low social threat). However, we anticipated that it would still possible to see a faster RT time to the boss when the boss was paired with others (not including the self, such as a labmate), due to his or her general social dominance over the labmate. This would demonstrate that the boss evokes a faster RT in general social situations, despite not directly impacting the individual's self-processing. The current study replicated the study from Ma and Han (2009) with European American participants to test these hypotheses. We report data from both the current study and the previous study [20] for cross-cultural comparisons.

## Methods

### Ethics Statement

This study was approved by the University of Southern California Institutional Review Board and by the local ethics committee in Beijing, China and was performed in accordance with the 1964 Declaration of Helsinki.

### Participants

Twenty healthy European American graduate students in America (10 females / 10 males, mean age of all participants = 26.6, SD = 3.05) and twenty healthy Chinese graduate students in China (10 females / 10 males, mean age of all participants = 24.8, SD = 1.94) participated in this study. All were right-handed and had normal or corrected-to-normal vision. In addition, all had worked with their advisors for more than one year (13–60 months), and advisors were of the same race as the student to avoid confounds due to the social influences of race. Written informed consent was obtained from all participants before inclusion in the study.

### Questionnaire measurement

Participants were given a modified Brief Fear of Negative Evaluation (Brief-FNE) scale [21] to assess their fear of being negatively evaluated by both their advisor and another faculty member who worked for the same department but was not in the participant's lab (e.g., I am afraid that Professor XXX will not approve of me). Participants used a 5-point Likert scale (1 = not at all characteristic, 5 = extremely characteristic) in response to each item, reporting how properly each statement fit them in respect to 1) their advisor and 2) the other faculty member. In addition, participants were asked to rate each professor's (advisor, other faculty member) social status, which was defined as the individual's ability to exert influence over other people and institutions, on an 11-point Likert scale (0 = not at all dominant, 10 = extremely dominant).

### Stimuli and procedure

Ten digital face images were taken from each participant, his/her faculty advisor, another faculty member, and one of his/her labmates prior to the experiment. Half of the faculty advisors and other faculty members were of the same gender as the participant, and half were of a different gender as the participant. Participants knew both the faculty advisor and faculty member for the same length of time. In addition, an advisor for one participant might be used as the other faculty member for another participant, so as to match perceptual features of the stimuli.

**Figure 1:**
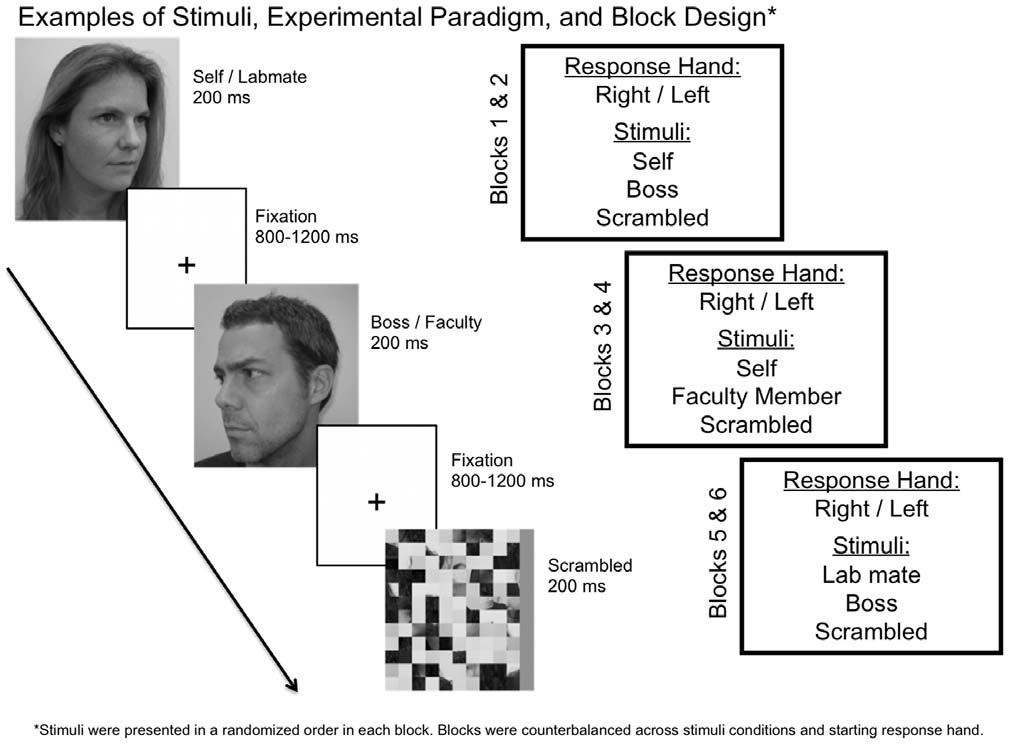
Examples of the stimuli, experimental paradigm, and block design. Participants were shown images of themselves/their labmate, their boss/faculty member, and scrambled images of faces for 200 ms, separated by a fixation cross that lasted between 800–1200 ms (left diagram). Blocks consisted of the following three stimuli sets (right diagram): self/boss/scrambled, self/faculty/scrambled, labmate/boss/scrambled, and were performed with both left and right hands, for a total of 6 blocks. Starting response hand and stimuli sets were counterbalanced across participants.

Five of the images of each individual were oriented to the left (varied from 30° to 90°) and the other five were oriented to the right. Participants were instructed to look directly ahead and maintain a neutral facial expression. Control images used scrambled images of the faces, which were created by dividing face images into 10×10 arrays and randomly rearranging them, using Matlab. These images were presented with a gray bar on either the left or the right. For an example of all stimuli and the experimental paradigm, see Figure 1. The participants in this figure have given written informed consent (as outlined in the PLoS consent form) to the publication of their photographs. All images were calibrated in luminance and contrast and subtended a visual angel of 2.13°×2.17° at a viewing distance of 70 cm. Images were presented for 200 ms each at the center of the screen, with a varying intertrial interval of 800 to 1200 ms during which a fixation cross was presented. Participants were instructed to indicate whether faces were oriented to the left or the right, or whether the gray bar of scrambled images was on the left or the right, by pressing two keys using the index and middle fingers. Task instructions emphasized both speedy and accuracy.

Each block of trials contained 40 face images and 20 scrambled images. The block design is illustrated in Figure 1. Self-face was presented in a high-threat context (20 trials each of self-face, advisor's face in each block) for two blocks and in a low-threat context (20 trials each of self-face, other faculty member's face in each block) for two blocks. In addition, two blocks used 20 trials of each a labmate's face and the advisor's face in order to discern whether the advisor's face generated increased processing speed when paired with non-self faces. For each stimulus condition, participants responded with the left hand in one block and the right hand in the other block. The order of responding hands and conditions was counterbalanced across participants.

## Results

### Subjective ratings

**Figure 2:**
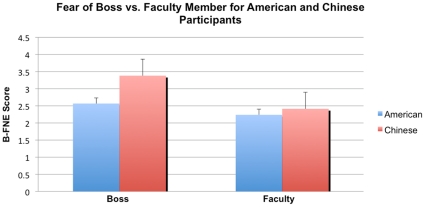
Chinese and American ratings of fear of negative evaluation from bosses versus faculty members. Participants ratings of fear of negative evaluation from the Brief-Fear of Negative Evaluation (B-FNE) questionnaire are presented for the boss (left; Americans in blue, Chinese in red) and for the other faculty member (right; Americans in blue, Chinese in red).

Both European American and Chinese participants' subjective reports indicated comparable perceived social status of their advisors and the other faculty members (European Americans: 5.90±2.29 vs. 6.0±1.89, t(1,19) = −0.276, p = 0.79; Chinese: 8.30±1.45 vs. 7.85±1.57, t(1,19) = 1.690, p = 0.107). In addition, the results of the Brief-FNE scale suggested that both European American and Chinese participants were significantly more afraid of negative evaluation from their advisors than from the other faculty members (European Americans: 2.56±0.44 vs. 2.24±0.39, t(1,19) = 3.482, p = 0.0025; Chinese: 3.38±0.73 vs. 2.41±0.66, t(1,19) = 5.265, p<0.001). However, a 2-factor mixed-effects analysis of variance (ANOVA) with Culture (Chinese, American)×Threat (Boss, Faculty Member) demonstrated an interaction effect between Chinese and American participants' reports of negative evaluation from their boss versus their faculty member (F(1,19) = 9.536, p = 0.004; see Figure 2), with Chinese participants reporting higher fear of negative evaluation from their bosses than European American participants.

### RT results

Response accuracy was high for both European American and Chinese participants in face orientation judgment tasks (European Americans: 97.42%±2.21%; Chinese: 94.96%±2.43%). RTs with correct responses and within three standard deviations were analyzed. As used by two of the authors in a previous study [18], RTs were normalized by dividing RTs to self/other faces by RTs to scrambled images to rule out the influence of difference in response selection and execution between different blocks. Response accuracies and normalized RTs were then subjected to repeated measure ANOVAs with Hand (left vs. right hand), Face (self vs. other faces), and Threat (high- vs. low-threat) as independent within-subject variables. Results from Chinese participants have been reported previously [20]. Thus, here we first report results from European American participants, followed by cross-cultural comparisons with results from Chinese participants.

### European American RT results

While none of the response accuracies showed significant effects (p>0.05), ANOVAs of normalized RTs showed a significant effect of Face (F(1,19) = 11.403, p = 0.003), with normalized RTs to one's own face being faster than RTs to other faces.

There were no significant interaction effects, and the finding of a Face×Threat interaction in Chinese subjects (F(1,19) = 58.469, p<0.001) [20] was not found with European Americans (F(1,19) = 1.911, p = 0.182), suggesting a comparable RT self-face advantage when self-face was presented with the boss and faculty member in Americans.

Normalized RTs to faces of labmates and advisors were also subjected to an ANOVA with Hand (left vs. right hand) and Face (labmate vs. advisor) as independent within-subject variables. While this analysis did not yield significant results in Chinese participants [20], it did yield a significant interaction effect between Hand and Face in European American participants (F(1,19) = 6.618, p = 0.018). A post-hoc analysis revealed that normalized RTs were significantly faster for the advisor's face on left-hand trials (0.88±0.148 vs 0.91±0.162; t(1,19) = 1.78, p = 0.045) but not on right-hand trials (0.90±0.174 vs. 0.91±0.180; t(1,19) = −0.32, p = 0.38).

### Correlation analysis

To assess whether subjective evaluation of social threat from others affected these behavioral performances associated with self-face recognition, we correlated mean ratings from the Brief-FNE scale related to advisors and the differential RTs (normalized RTs to self-face minus normalized RTs to advisor's face). We did not find any significant correlations between either left, right, or combined hand responses and these scores (ps>0.05).

**Figure 3:**
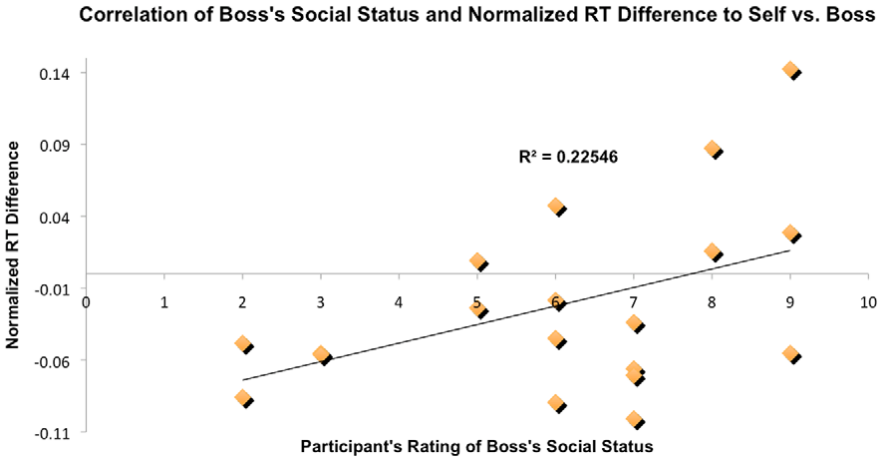
Correlation between boss's perceived social status and normalized RT difference in European Americans (boss-self). Participants' ratings of their boss's social status (x-axis) correlates positively with normalized RT differences (self minus boss; y-axis), R^2^ = 0.225, p = .034.

We then assessed whether subjective ratings of perceived social status correlated with differential RTs (normalized RTs to self-face minus normalized RTs to advisor's face). We found a significant positive correlation between boss's perceived social status and left-hand responses (r = 0.475, p = 0.034), as shown in Figure 3. This effect was not found for right-hand responses (r = 0.282, p = 0.228). Additionally, this effect was not found when correlating the social status of the other faculty member with differential RTs (normalized RTs to self-face minus normalized RTs to other faculty member's face) for either hand (ps>0.05).

### Cross-Cultural RT results

To assess differences between European American and Chinese participants, a mixed-design ANOVA was assessed with Culture (European American vs. Chinese) as a between-subjects factor, and Hand (left vs. right hand), Face (self vs. other faces), and Threat (high- vs. low-threat) as independent within-subject factors.

The four factor ANOVA revealed a marginally significant interaction effect of Culture×Face×Threat (F(1,19) = 3.616, p = 0.073), as the interaction of Face×Threat was more salient in Chinese subjects (F(1,19) = 58.469, p<0.001) than in American subjects (F(1,19) = 1.911, p = 0.182). There was also a significant interaction effect of Culture×Face (F(1,19) = 12.409, p = 0.002), with faster normalized RTs to one's own face in European Americans (F(1,19) = 11.403, p = 0.003) than in Chinese participants (F(1,19) = 0.712; p = 0.409).

**Figure 4:**
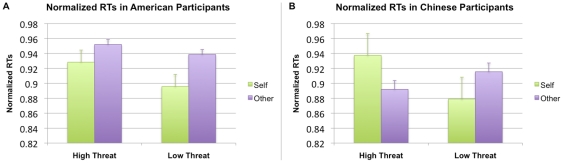
Bar graphs depicting Culture×Face×Threat normalized RTs (left hand only). American participants demonstrated a self-face advantage in both high threat (self and boss) and low threat (self and other faculty member) blocks shown on the left (A). Chinese participants demonstrated a boss-face advantage in the high threat block (self and boss), but a self-face advantage in the low threat block (self and other faculty member), shown on the right (B).

Given prior findings suggesting that the self-face advantage has a more significant effect on left-hand responses [15], [16], [18], [20], we then analyzed data from left-hand responses. Using left-hand responses only, we found a significant interaction between Culture×Face×Threat (F(1,19) = 7.003, p = 0.018). As demonstrated in Figure 4, while the normalized RTs were significantly faster to the self in the high-threat condition for European Americans, normalized RTs were significantly faster to boss in the high-threat condition for the Chinese participants. This pattern of self-face advantage persisted in European Americans during the low-threat condition, while Chinese participants regained self-face advantage during the low-threat condition.

## Discussion

The current study examined how cultural differences in self-construal affect one's implicit self-processing in different social contexts. We compared normalized RTs of American and Chinese participants during an implicit face orientation task and discovered that, while both groups show a self-face RT advantage when self-face was presented with a faculty member's face (low-threat condition), only Chinese participants showed a loss of self-face advantage, replaced with a boss-face advantage, when self-face was presented with the boss's face (high-threat condition). In contrast, American participants maintained a self-face RT advantage in both low and high threat conditions, in accordance with our hypothesis that self-processing in Americans is not influenced by the social threat of one's boss. Interestingly, the correlation results show a modulation of this effect in Americans by their boss's perceived social status, so that the self-face advantage decreased as the subjective feelings of the boss's social status increased. Overall, these results demonstrate that culture modulates how self-processing is affected by the presence of a social threat and that the very concept of a “boss” may hold vastly different meanings in different cultures (i.e., negative threat in interdependent cultures versus social dominance in independent cultures).

### Cultural Selves and Social Threats

The results of the questionnaire measurements suggest that both European American and Chinese participants reported significantly greater fear of negative evaluation from their advisor than from another faculty member, despite giving comparable ratings of social status to both advisors and faculty members. This suggests a culturally universal pattern in which advisors, who have direct influence over our participant pool of graduate students, constitute a greater social threat than other faculty members, despite equal social status. However, the ratings were greater overall in Chinese participants, suggesting that Chinese participants are more likely to fear negative evaluation from their bosses than American participants. This is in line with the idea that interdependent self-construals are more sensitive to fear of negative evaluation from others than independent self-construals [5], [22] and holds implications for multicultural work environments in which individuals may be more or less sensitive to different forms of evaluation and feedback from their supervisors, depending on their cultural self-construals [22]–[24].

In line with this finding, European Americans' self-face advantage was not diminished by the presence of their advisors during the high-threat conditions, as was found in Chinese participants. Instead, European American participants had faster RTs to their own face in both low-threat (self and faculty member) and high-threat (self and advisor) conditions, maintaining the self-face advantage regardless of social context. Numerous studies on Western versus East Asian culture have associated East Asian culture with greater collectivism and attention to context and Western culture with greater individualism and attention to focal points [25]–[29]. Our results correspond with these prior findings, suggesting that Westerners are less influenced by the presence of social context (e.g., the other faces in the block) than East Asians during a self-face recognition task. This may be due to the robustness of European American's self-concept, which is individualistically defined, as compared to the holistic representation of self found in Chinese participants, which often takes into account not only the self but also others within one's social circle [8], [9].

### Cultural Variations of the Boss

While European American participants did not show a boss-face advantage, faster RTs to the boss's face compared to their own face were correlated with the boss's perceived social status and relative social influence. That is to say, advisors with higher perceived social status had a stronger effect on participants' self-face response than advisors with lower social status. This is in stark contrast to Chinese participants, all of who showed a loss of self-face advantage during high-threat blocks, regardless of the boss's social status. Interestingly, in Chinese individuals, faster RTs to the boss's face compared to their own face were correlated with how much they feared negative evaluation from their boss. Thus, in both cultures, faster RTs to the boss's face compared to self-face were correlated with a behavioral measure—but these measures are very different. For Chinese participants, it was fear of negative evaluation from the boss, while for Americans, it was the boss's social dominance.

These findings lead us to suggest that the very concept of the “boss” holds different social meanings in independent versus interdependent cultures. Namely, the boss may represent a social threat related to the fear of negative evaluation in more interdependent cultures, particularly where there are more set, hierarchical relationships with greater “power distances” between positions [11], [12], [22]. In contrast, in cultures with more independent self-construals and less distance between the levels of power of the boss and the employee [22], the boss may represent varying degrees of social dominance, which is dependent upon his or her perceived social status. It appears that one's cultural conceptualizations of oneself mediate this attitude, as Americans tend to focus on primary control, emphasizing autonomy, the self-made man, and personal goals above work goals, while the Japanese focus on secondary control, emphasizing teamwork, the good of the team above all else, and distinct hierarchical levels [12]. This is also reinforced by the neuroimaging finding that mesolimbic reward regions in the caudate nucleus and the medial prefrontal cortex are active during observation of signals related to social dominance for Americans and social subordination for Japanese participants, indicating that cultural differences in social attitudes are personally rewarding [13].

In addition, these findings are in accordance with Ma & Han's (2010) implicit positive association (IPA) theory of self-face advantage. In one of their experiments, they demonstrated that both Americans and Chinese participants showed an elimination of the self-face advantage after negative threat-to-self-concept priming, although Americans did not demonstrate as great a decrease in self-face advantage as Chinese participants, suggesting that Americans were more robust to threats to self [18]. Here, we showed that Americans do not demonstrate a loss of self-face advantage in the presence of the boss, suggesting that the boss does not constitute a threat to oneself for American participants, while it does for Chinese participants, who did lose the self-face advantage in the presence of the boss.

While the boss may not constitute a direct negative threat to one's self-concept in American participants, thus not eliminating one's self-face advantage, it is notable that American participants *did* demonstrated faster responses to their boss when his or her images were presented with a labmate's images. This indicates that there may be an effect of one's boss in more general social settings, which are not found in self-related settings. Notably, this reaction was only found in left-hand blocks, which suggests a lateralization of the effect to the right hemisphere, which has been associated with emotional communication [30], [31] and social behavior and social interactions [32], [33]. Thus, while one's social superior may not necessarily influence American participants' self-perception, he or she may affect more general social situations in which social dominance plays a role. Under this line of reasoning, it follows that the presence of one's boss might affect overall social perception, such as having a faster reaction to someone who is more socially dominant (boss) than someone who is socially inferior (labmate). Altogether, these findings shed light on the role that a social superior may play in different cultural settings. While a boss may constitute a social threat in interdependent cultures, it appears that a boss represents general social dominance in more independent cultures, a finding that holds significant consequences for cross-cultural relationships, both in the workplace and beyond.

### Future Directions

While the current study examined cross-cultural behavioral effects on self-face recognition in social contexts, future studies may explore the neural mechanisms underlying these observed effects and how culture modulates the related neural activity. Prior research suggests that several brain regions may be involved in these processes, namely in the medial prefrontal (mPFC) [8], [9], [34], the right prefrontal cortex [35], and the right parietal cortices [19], [36]. Cultural neuroscience studies of self-traits [8], [34] and self-face recognition [35] indicate that the medial prefrontal cortex, which is involved in self-representation and tends to be more active in response to oneself than to others during trait judgments, also represents close and familiar others (e.g., one's mother) in East Asian cultures but not in Western cultures. In addition, this effect can be modulated by culture-specific priming, with priming towards more interdependent ideals enhancing the representation of close others in the mPFC and priming towards more independent ideals decreasing mPFC activity [34]. Applied to the current study, it is possible that strong neural representations of the self in brain areas such as the mPFC in American participants stand against the influence of social contexts to a greater degree compared to Chinese participants, thus not demonstrating a boss-effect on the typical self-face advantage. In addition, the right parietal region has also been implicated in self-other distinctions, as shown by repetitive transcranial magnetic stimulation to the right parietal cortex disrupting performance in a self-other face recognition task [36] and an fMRI study demonstrating right hemisphere activation in the parietal, frontal and occipital regions during self-face recognition [19]. These results are consistent with our findings of a stronger effect on left- than right-hand responses, and suggest that the right parietal region may also play a role in the cultural modulation of this effect. Finally, as discussed by Ma and Han (2010), emotion-related regions, such as the anterior cingulate and anterior insula, may also affect self-versus-boss representations [18]. Future neuroimaging research may help to better understand the neural regions responsible for these sociocultural effects.

### Conclusion

The current study demonstrated the strong effects of culture on self-processing in the presence of one's social superior. Not only do these results reveal the ways in which self-construals are affected by social threats, with interdependent self-construals more strongly influenced than independent ones, but they propose that what constitutes a social threat may differ across cultures. Specifically, we suggest that the concept of a “boss” may hold vastly different meanings for individuals from East Asian versus Western cultures, representing a personal social threat in the former and general social dominance in the latter. Research on cultural differences has already noted that cultural tendencies (e.g., collectivist vs. individualist; high vs. low power distance) have an impact on leadership behavior [22], [37], political communication [38], career counseling [39], work team dynamics [40], and investments and economic outcomes [41], to name a few. Future research may explore whether and how cultural differences in relation to one's social superior present themselves in the workplace and political arenas, and whether there are ways to effectively mediate these differences, as one study has suggested that the self demonstrates cultural plasticity and can be modulated by cultural priming [42]. These findings are particularly salient as globalization increases, and along with it, the prevalence of multicultural work, political, and public environments, particularly between East Asian and Western partners. Studies of multicultural workplaces indicate intricate dynamics between in- and out-group members based on their individualistic/collectivist tendencies [43], [44], communication styles, and interpersonal relationships [23], [24], [45]. Better understanding of cross-cultural social relationships and social hierarchies may elucidate the ways in which individuals hold different culture-based social understandings and expectations. These enhanced understandings may in turn help to foster smoother and more productive global collaborations and exchanges.
